# Clinical artificial intelligence—the case for a new physician role

**DOI:** 10.1016/j.lana.2025.101280

**Published:** 2025-10-25

**Authors:** Arjun Mahajan, David W. Bates

**Affiliations:** a Harvard Medical School; Boston, MA, USA; b Brigham and Women's Hospital; Boston, MA, USA

Artificial intelligence (AI) is rapidly moving from experimental settings into clinical practice. Large language models, multimodal systems, and predictive algorithms are applied to diagnosis, triage, documentation, and decision support.^1^ These systems promise efficiency and reach. Yet they also introduce new complexity. Clinicians are asked to interpret, apply, and monitor tools they did not design, often with little training in how such models function.

Medicine has encountered similar transitions before.^2^ The adoption of imaging technologies led to the creation of radiology. The rise of molecular assays created clinical genetics. The use of tissue microscopy shaped modern pathology. Each field had roots as a technical adjunct, then matured into a clinical discipline with distinct expertise, training pathways, and standards of practice—driven by factors beyond the technology itself. The question is whether artificial intelligence now requires the same professionalization.

AI differs from earlier medical technologies in several ways: in particular, it is not static.^3^ Models evolve as data accumulate, environments shift, and updates are deployed. Without oversight, clinicians may not recognize when performance drifts, when training data are unrepresentative, or when outputs conflict with goals.

The case for a clinical AI physician is not about coding—rather, it is about stewardship. Specialists in clinical AI would understand the logic of model development, the limitations of validation, and the standards required for safe implementation. They would mediate model-aided care, ensuring outputs are contextualized, uncertainties communicated, and updates monitored.

Skeptics may argue all physicians can acquire this literacy. Yet the volume and technical depth of emerging tools suggest otherwise.^4^ Just as not every physician interprets a biopsy, not every physician may be equipped to adjudicate the reliability of a complex model. Consider a physician choosing between lung cancer prediction models: one platform performs best with genetic data, another with imaging, another in select demographics—highlighting the complexity of navigating manifold technical considerations. Conversely, while non-clinicians can certainly design and validate models, physicians uniquely understand how outputs intersect with clinical contexts, systems of care, and patient preferences.

The path forward need not be rigid: clinical AI may emerge as a distinct specialty or as an expanded pathway within existing subspecialties, such as clinical informatics.

Current clinical informatics training spans a range of competencies such as health information technology, data interoperability, and system-level decision support implementation.^5^ Yet the clinical application of AI requires a related but distinct set of capabilities — including validation literacy (interpreting performance across diverse populations and contexts), drift surveillance (ongoing monitoring for model degradation), change-management under planned change control pathways for adaptive algorithms (PCCPs), and risk communication with patients and care teams.^1^^,^^6^^,^^7^ Training might emphasize the applied use of AI insights within care teams, a role more akin to radiology or pathology consultation than system implementation. In this way, clinical AI training would complement or expand on, rather than duplicate, existing informatics training structures. Given that AI systems evolve rapidly, training would likely need to emphasize principles of critical appraisal and continuous learning rather than mastery of any one platform. From an employment perspective, health systems, payers, and regulators all have incentives to ensure these functions are present, since oversight of AI carries implications for liability, reimbursement, and safety.

Whether these functions ultimately coalesce into a distinct specialty or evolve as a track within existing training pathways remains an open question, though one that may echo how new competencies have historically found their footing in medicine. What matters now is recognizing that medical AI is not a fixed tool but an evolving practice requiring interpretation and oversight. Assigning responsibility is necessary for accountability.

Deciding who is prepared to train, monitor, and interpret these systems is no longer a theoretical exercise; it is a matter of patient safety and professional duty.

## Contributors

AM: conceptualization, methodology, formal analysis, writing—original draft. DWB: conceptualization, methodology, supervision, formal analysis, and writing—reviewing and editing.

## IRB approval status

Not applicable.

## Patient consent

Not applicable.

## Declaration of interests

AM has no declared conflicts of interest. DWB reports grants and personal fees from EarlySense, personal fees from CDI Negev, equity from ValeraHealth, equity from Clew, equity from MDClone, personal fees and equity from AESOP, personal fees and equity from Feelbetter, equity from Guided Clinical Solutions, and grants from IBM Watson Health, outside the submitted work. DWB has a patent pending (PHC-028564 US PCT), on intraoperative clinical decision support.
